# Statistical evaluation of the achievements of professional students by combination of the random forest algorithm and the ANFIS method

**DOI:** 10.1016/j.heliyon.2023.e21768

**Published:** 2023-10-31

**Authors:** Marija Mojsilović, Radoje Cvejić, Selver Pepić, Darjan Karabašević, Muzafer Saračević, Dragiša Stanujkić

**Affiliations:** a Academy of Professional Studies Sumadija Department in Trstenik, Trstenik, Serbia; b Faculty of Information Technology and Engineering FITI, University Union Nikola Tesla, Belgrade, Serbia; c Faculty of Applied Management, Economics and Finance, University Business Academy in Novi Sad, Belgrade, Serbia, Belgrade, Serbia; d Department of Computer Sciences, University of Novi Pazar, Novi Pazar, Serbia; e Technical Faculty in Bor, University of Belgrade, Bor, Serbia

**Keywords:** Artifical intelligence, Anfis, Random forest, Computational intelligence, Education

## Abstract

This research is of great importance because it applies artificial intelligence methods, more specifically the Random Forest algorithm and the Anfis method to research the key factors that influence the success of students in vocational schools. Identifying these influencing factors is not only useful for improving curriculum and practice but also provides valuable guidance to help students master the material more effectively. The main goal of this research is to penetrate deeply into the core of the factors that influence the success of students in vocational schools, using two different methods. Each of the factors represented as input is mutually independent and does not affect each other, but each of them affects the output variable. The parameters considered as input variables are prior programming knowledge and pretest requirements. Then, by finding one factor that has the greatest influence, the factor of pre-exam obligation was investigated in more detail, using the Anfis method, which was broken down into several input parameters. These results emphasize the importance of the combination of the Random Forest algorithm and the ANFIS method in the statistical evaluation and assessment of student achievement in vocational schools. This study provides useful guidelines for improving education and practice in vocational schools to optimize educational outcomes.

## 1 Introduction

Education is crucial for the personal and professional development of an individual, and its main purpose is to facilitate the process of acquiring knowledge and skills that are essential for a successful future life and career. Mastering the material and achieving academic goals is an essential part of the educational process. However, achieving these goals is not always easy, and many factors can affect students' ability to absorb material effectively.

This research focuses on the input factors that play a significant role in improving students' ability to master the material. Understanding these factors is crucial, as it allows educational institutions, teachers, and students to recognize and adequately respond to the challenges they face during learning.

In the last few decades, numerous studies have addressed these key questions and laid the foundation for understanding the impact of input factors on students' academic performance, using a variety of methods. This literature review aims to synthesize and analyze previous works and research that have dealt with this topic, identifying key conclusions and trends in the area.

Through the analysis of relevant studies and papers, the research will focus on the identification of key input factors that have been investigated in the context of improving students' ability to master the material. These studies will lay the foundation for further research to understand how these factors feed into each other and how they can be adequately addressed to achieve better academic performance. This literature review is essential to the development of educational strategies, policies, and practices that will support students in their learning and create successful educational experiences.

The explosive growth of the application of artificial intelligence has an increasing application in all areas, including education [[1], [2], [3], [4], [5]]. The application of artificial intelligence technologies, such as intelligent training systems and robots, supports and improves education [6]. The application provides great potential for improving learning, teaching, and assessment, encouraging teachers to understand students and a more accessible way of learning to better master the material [[7], [8], [9], [10], [11]].

Artificial intelligence refers to a digital machine that can perform tasks related to computer vision, speech, machine learning, big data processing, and the like [[12], [13], [14]].

Random Forest is one of the popular machine learning algorithms that can be applied to various problems, and it is characterized by its flexibility and ease of use [15]. This algorithm builds a forest, which consists of a series of decision trees. Each tree is trained on a randomized subset of the data and a better result is obtained by combining them. Using the Random Forest algorithm improves performance over single decision trees, making it a popular choice in the field of machine learning [16].

By using neural networks, it is possible to form a model of the fuzzy inference system, which is used to calculate the parameters of the membership function based on the available input-output data. This model is defined based on available knowledge about the process being studied. The ANFIS network algorithm passes through four layers, and during the first pass, the signals move forward through the network. In the fourth layer, the method of least squares is used to determine the optimal parameters [[17], [18], [19]].

“Fuzzy logic is a popular logical system used in many applications in real life” [20]. The papers [21,22] present the application of neural network artificial intelligence, i.e. the Adaptive Neuro-Fuzzy Inference System (ANFIS) methodology as support for the improvement of mathematics teaching.

Random Forest algorithm is widely used in prediction and evaluation in the education field which is presented in the papers [[23], [24], [25], [26]].

Today, an increasing number of vocational schools aim to prepare their students for successful careers in various fields, as there is a decreasing interest among students it is important to research and identify factors to help teachers and students achieve better results.

In the following, several key factors that can be associated with the success of students in vocational schools will be considered, taking into account both individual and social factors that influence their progress. By obtaining the factors, it is easier for the teacher to design the teaching, and for the student to focus on the factors that have the greatest impact on achievement. The organization of the work that is presented further in the paper consists of hypotheses, goals, tasks and methodology, then the results and discussion obtained by the random forest algorithm, as well as the results and discussion obtained by the anfis method and the conclusion.

## 2 Hypotheses, goals, tasks and methodology

The subject Introduction to Programming at the Academy of Vocational Studies in Šumadija, Trstenik Department, aims to provide students with knowledge of advanced programming techniques through examples in the C language. Exercises for creating tasks are performed in the CodeBlocks program. The basis of programming consists of a methodology of approach to solving tasks with the help of computers. This methodology includes problem analysis and definition of a mathematical model, selection of a numerical solution method, algorithm design and definition of data structure and programming language, program editing, testing and error correction, and more. This approach to programming enables students to successfully use advanced programming techniques and is especially important for students who are in professional studies.

This research, related to the detection of factors for the improvement of teaching in the subject Introduction to Programming, was conducted at the Academy of Professional Studies Sumadija – College in Trstenik. The data were collected during two school years in the first year of professional studies attended by 212 students. Data were collected as official school statistics, as well as through a survey conducted among students.

The goal of the research is to use the artificial intelligence method, that is, the Random Forest algorithm and the ANFIS method, to detect the factor that most affects the outcome, that is, the total number of points on the student's final exam.

The research tasks are:

• Using the Random Forest algorithm, determine which of the two selected factors has a greater influence on the student's final grade
• Using the ANFIS method, determine which factor has the greatest influence on the student's grade, with a more detailed analysis of the factor obtained in the first part of the research.

The main hypothesis, which is expected that the Random Forest algorithm and the ANFIS method will predict the factors that most influence the achievements of students in the subject Introduction to Programming, is operationalized on two hypotheses:

Hypothesis 1 It is assumed that the analysis with the Random Forest algorithm will show that the pre-exam tasks have a greater influence on the student's achievements in the final exam than their prior knowledge.

Hypothesis 2 It is assumed that the analysis using the ANFIS method will show that the first colloquium or the second colloquium has the greatest impact on the student's achievements in the final exam.

## 3 Results and discussion obtained by the random forest method

This part of the research deals with the assessment of which factor has a greater influence on the achievement of students in the subject Introduction to Programming. As a parameter to be studied, the knowledge achieved in the final exam is taken, which is coded as:

• 0-poor knowledge
• 1-intermediate level of knowledge
• 2-high level of knowledge

This indicator relies on two independent variables that were used in the research:

• Prior knowledge of programming - represents whether students previously studied programming in secondary schools, it is a self-assessment of students about their prior knowledge of programming in an interval from 0 to 10
• Pre-examination requirements - which represent the number of points the student earned during the semester, ranging from 0 to 70.

Prior programming knowledge can have a significant impact on the final programming grade. Students who have more prior knowledge in this area may have an advantage in understanding new material and mastering new areas faster. Also, students with more prior knowledge may be able to focus on more complex and deeper levels of material, while those with less prior knowledge may have difficulty keeping up with the lessons and facing new areas. However, it is important to note that prior knowledge is only one factor that affects the final grade and that other factors, such as engagement in classes and studying, which together make up the pre-examination requirements, also have a great impact on student achievement.

The sample in this research consists of students in the first year of undergraduate studies, the data were collected during two school years, and 212 students participated in the survey.

**Fig. 1:**
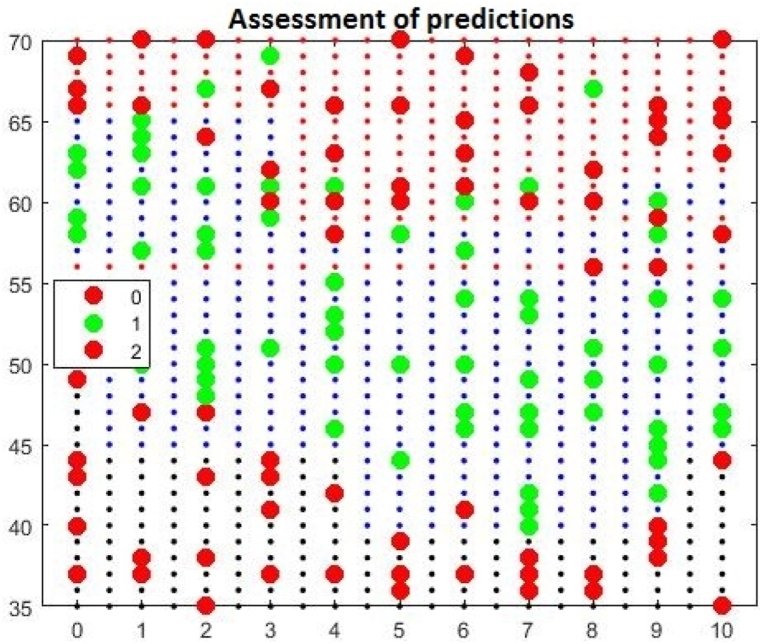
Prediction evaluation chart.

Research using the Random Forest algorithm visually displays the results, which are shown in the diagram, Fig. 1. Students with high points on the pre-examination requirements reach a high level of knowledge when the red dots on the diagram reach the final level, which is the maximum 70 points. This graphic display makes it easier to recognize the influence of pre-examination requirements and prior knowledge of programming on the final academic results of students. Students who have prior knowledge of programming and who have a high number of points on the pre-examination requirements achieve a high level of knowledge, that is, the maximum number of points on the final exam. There are deviations in the model that occur due to the random distribution of the data set, but algorithm optimization can help resolve them.

**Fig. 2:**
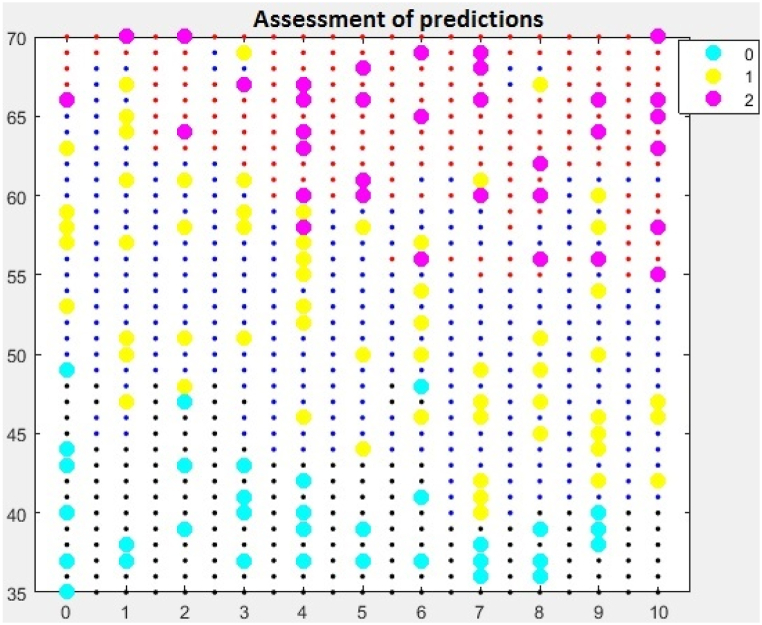
Prediction evaluation chart.

If no optimization is performed, the "fit-ensemble" function can be used. This function is intended to adjust the learner set for classification problems and regression. It already provides a realistic assessment that students who achieved high points on the pre-examination requirements and showed activity in class, received high grades, Fig. 2.

Based on the presented research, it can be concluded that by predicting the factor that has the greatest influence on student achievement is the pre-examination obligation factor. The results showed that students who devoted themselves more to the pre-exam obligation tended to achieve better grades. Therefore, it is recommended that students be encouraged to regularly check their knowledge through colloquiums, class exercises, and homework, to improve their achievement of better results.

In this research, the Random Forest algorithm was applied to identify the factor that most influences student achievement. This research has shown that pre-examination requirements are a key factor affecting student achievement. The conclusion is confirmed by earlier research that also pointed to the importance of pre-exam duties in achieving good results on the exam. However, this research goes a step further, using the Random Forest algorithm to identify the factors that most influence student achievement.

These results have practical implications for the educational system and the vocational education system. For example, schools could focus on providing additional support to students in terms of exam preparation and pre-examination tasks to improve their exam performance.

Additionally, the results highlight the importance of the Random Forest algorithm in identifying factors that influence student achievement. Additional research would confirm the importance of continuous monitoring of learning and the development of strategies that can help increase the commitment and success of students in a professional environment, and therefore further research is being conducted in the form of a breakdown of the obtained factor that most affects student achievement, namely the student's pre-examination obligations.

Using another method, which is the Anfis method, leads to the identification of other key factors that influence the student's achievement, which are part of the pre-examination obligations, which could have important indications for the development of the educational system.

## 4 Results and discussion obtained by the anfis method

During the semester, students have pre-examination obligations consisting of colloquiums, seminar papers, projects, homework, and other activities. These assignments play an important role in preparing students for the final exam because they help them acquire the knowledge and skills they need. Regular performance of these duties helps students to better prepare for the final exam because through work on projects, seminars and other activities, they can check their knowledge and identify areas in which they are weaker.

The number of points for a student's passing grade is 51, and the maximum number of points on the exam is 100 and is used as an indicator of the achievement of students in the course Introduction to Programming during one semester. This indicator represents a reliable value that depends on many different independent variables, which are part of pre-examination requirements, as shown in Table 1. Those independent variables are:

**Table 1:** Input and output factors.

Inputs and Output	DESCRIPTION OF PARAMETERS	min - max
Input 1	class attendance	0,16–0,86
Input 2	independent work	0,1–0,9
Input 3	first colloquium	2–7
Input 4	doing homework	1–6
Input 5	second colloquium	2–7
Input 6	computer skills	6–7
Output	*number of points on the final exam (grade)*	*51–100*

Class attendance - represents the percentage of lectures and exercises that the student attended during the semester, the student could not attend classes and would be graded with 0 = 0 % attendance, on the other hand, he could attend all classes which entails 1 = 100 % attendance.

Independent work - the student's self-assessment of the share of learning time in the C programming language, to the total learning time, expressed as a percentage, the student can spend 100 % of his time learning programming, and he can spend 0 % of his time spent on programming.

In the first colloquium - the student does three tasks with subtasks, where he practically creates seven examples and can have from none to seven correct tasks. Depending on the number of tasks, the professor evaluates it with coefficients from 0 to 7.

Completion of homework - represents the number of assigned homework assignments completed by the student during the semester, out of 6 assigned homework assignments, the student can complete all 6 or none, i.e. 0.

Second colloquium - the student does three tasks with subtasks, where he practically creates seven examples and can have from none to seven correct tasks. Depending on the number of tasks, the professor evaluates it with coefficients from 0 to 7.

Competences for working on a computer - represents students' self-assessment of the level of their abilities and skills for working on a computer, in an interval from 1 to 10.

Research related to the detection of factors for the improvement of teaching in the subject Introduction to Programming was conducted at the Academy of Vocational Studies in Šumadija - Trstenik Department. The data were collected as official statistical data of the school and through a survey that was conducted during two school years, the research sample, that is, the respondents are first-year students attending the course on which the survey was conducted. Table 1 one shows the range of values that are exported to Matlab through a CSV document to obtain a result using the Anfis method. Table 1 shows the input parameters, with their minimum and maximum values, and the output parameter..

**Table 2:** The impact of one input on the output - the student's achievement.

Input	The input with the least error
One Input	Input no. 2
	TRAINING – ERROR
	SV = 0.000000 SD = 0.448215 MSE = 0.199774 **RMSE** = 0.446961
	TEST – ERROR
	SV = 0.069558 SD = 0.391378MSE = 0.153228 **RMSE** = 0.391443
	ALL DATA
	SV = 0.010549 SD = 0.43991 MSE = 0.19272 **RMSE** = 0.43899
Relibility of the model	Training data: R = 0.94326
	Test data: R = 0.9558
	All data: R = 0.94496

Further research using the Anfis method is shown in Table 2, where the mean value of the error, the mean deviation, the mean square error and the root mean square error for training data and test data are presented, as well as the reliability coefficient of the obtained model for the input that has the greatest impact on output size.

Input 2 has the smallest RMSE (root mean square error), which means that independent work has the greatest impact on the output size, that is, on the number of points the student has earned during the semester, which is the measure of student achievement. Given that Introduction to Programming is a subject that requires continuity in work, it is logical that independent work has a decisive influence on their achievement.

**Fig. 3:**
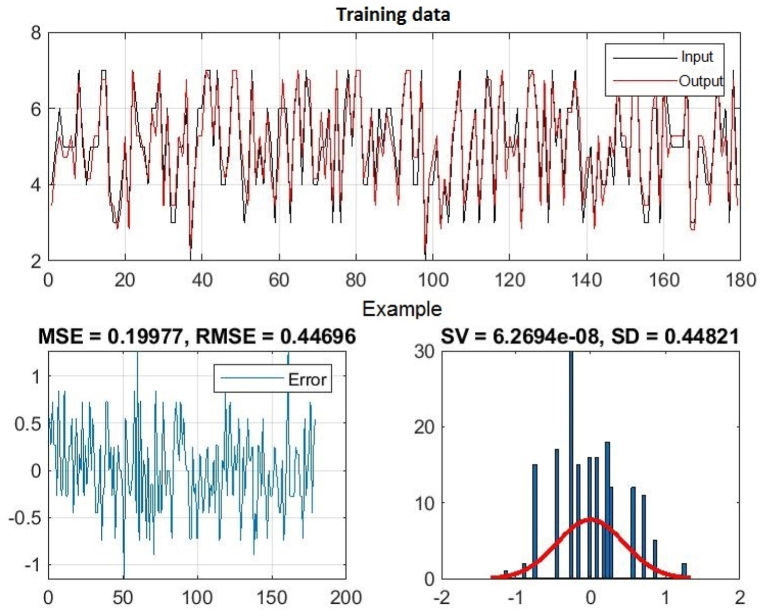
ANFIS network training - one input – achievements.

**Fig. 4:**
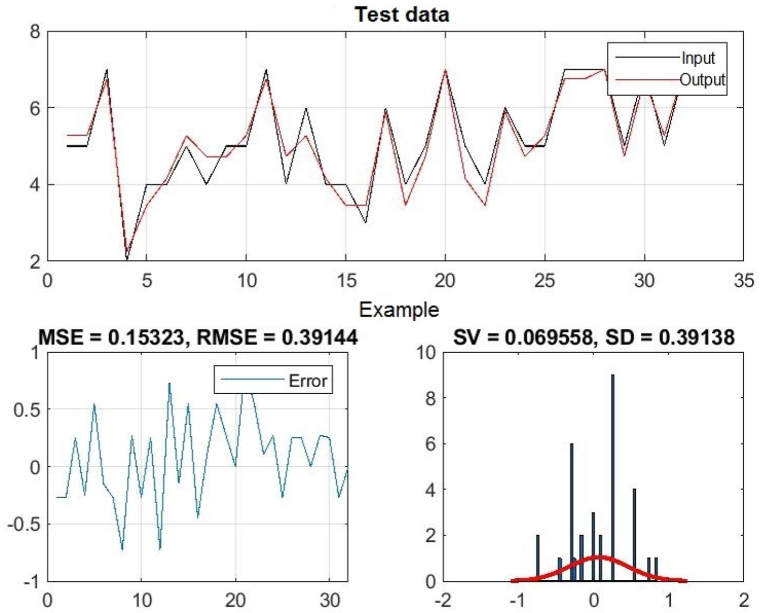
ANFIS network test - one input - achievements.

**Fig. 5:**
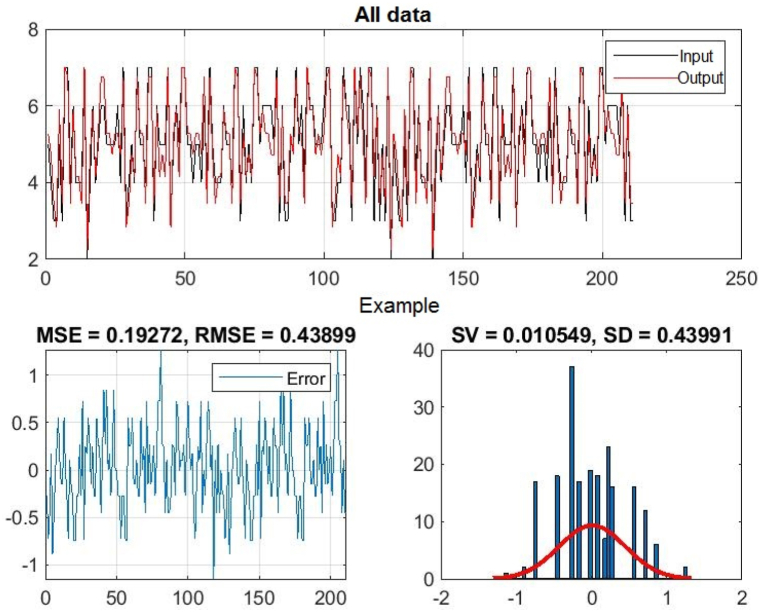
All data of the ANFIS network - one input.

**Fig. 6:**
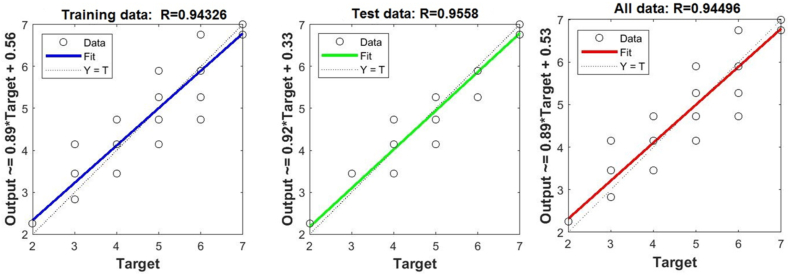
Regression of training, test and all data - one input – achievement.

**Fig. 7:**
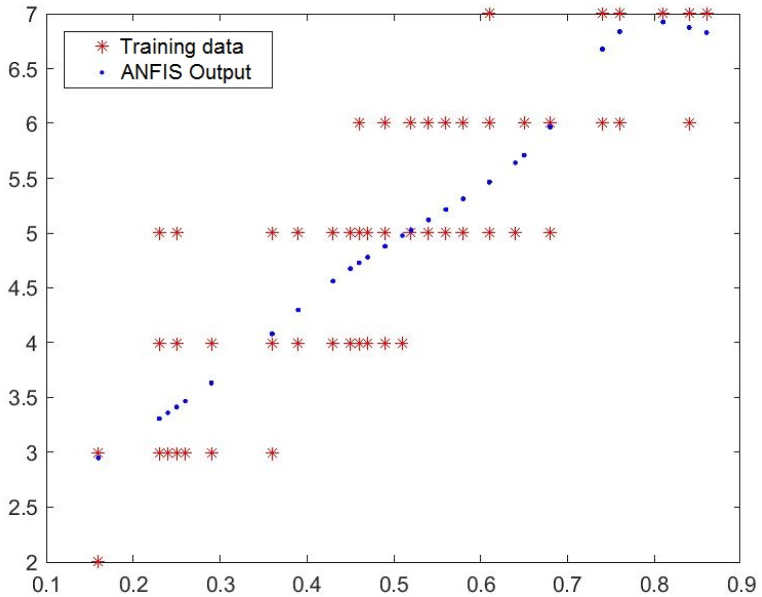
Graphical interpretation of training data - one input - achievements.

In Fig. 3, Fig. 4, Fig. 5, you can see the training, test and all data on errors in the training and testing process, which were obtained in the Matlab software package, using the ANFIS methodology. Fig. 6 shows the regression analysis and reliability of the model, and Fig. 7 presents a graphical interpretation of the training data.

In Fig. 3, in the upper part of the image, the graph of Training data with the output of one achievement factor is shown, while in Fig. 4, in the upper part of the image, the graph of Test data is shown, as well as in Fig. 5, the graph of All sub-data. In the lower left corner, Fig. 3, Fig. 4, Fig. 5 show a graph with the resulting root mean square error MSE and root mean square error RMSE. While in the lower right corner, the graph of obtaining the results of the mean value of the error SV and the mean deviation SD is shown.

Fig. 6 shows the linear correlation coefficient - R for Training, Test and All data. The linear correlation coefficient measures the degree of linear relationship between the values of the output variables collected during training and the output variables applied in testing. This coefficient, denoted as R, indicates the reliability of the model. Its value varies in the range from 0 to 1. The model is considered reliable when the value of the linear correlation coefficient is greater than 0.8.

In addition to the obtained one, the biggest impact of input on output, an assessment of the impact of two factors that have the greatest impact on output was also carried out. It was found that input 1 and input 2 in combination have the smallest RMSE, i.e., the biggest impact on the output size, which means that attendance at classes and exercises and time spent in the independent study have the most influence on the final number of points, i.e. the grade at the end of the semester. Independent work can further improve a student's performance, in addition to regular attendance.

The values of all errors of training, test, and combined data for the two most influential inputs together, input 1 and input 2, on the output size, as well as the reliability coefficient of the obtained model are presented in Table 3:

**Table 3:** The impact of two inputs on output - student achievement.

Input	The input with the least error
Two Input	Input no. 1 - 2
	TRAINING – ERROR
	SV = 0.000001 SD = 0.398426 MSE = 0.157856 **RMSE** = 0.397311
	TEST – ERROR
	SV = 0.004914 SD = 0.450383 MSE = 0.196530 **RMSE** = 0.443318
	ALL DATA
	SV = 0.00074619 SD = 0.40559 MSE = 0.16372 **RMSE** = 0.40463
Reliability of the model	Training data: R = 0.95297
	Test data: R = 0.95351
	All data: R = 0.95351

**Fig. 8:**
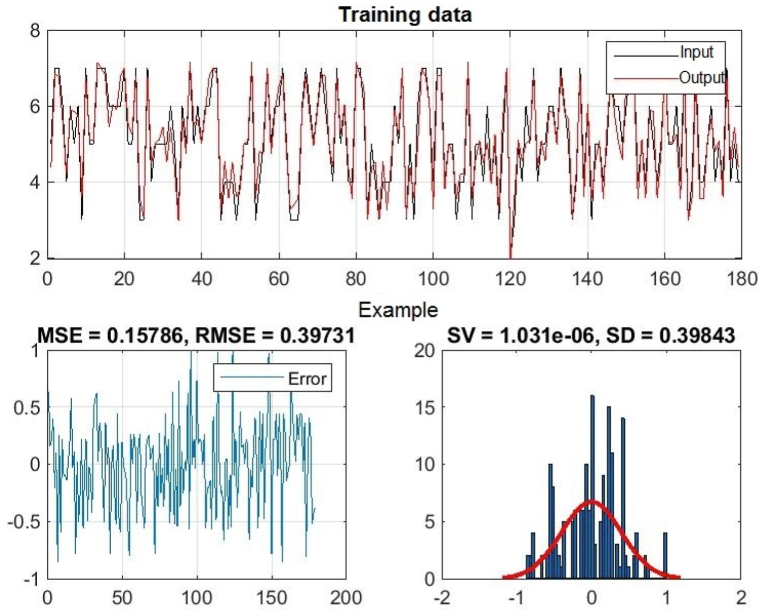
ANFIS network training - two inputs – achievements.

**Fig. 9:**
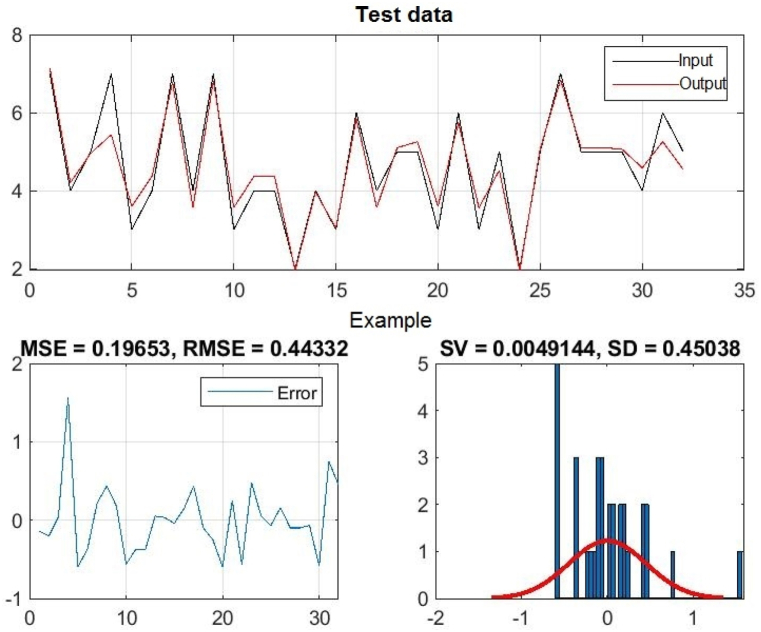
ANFIS network test - two inputs – outputs.

**Fig. 10:**
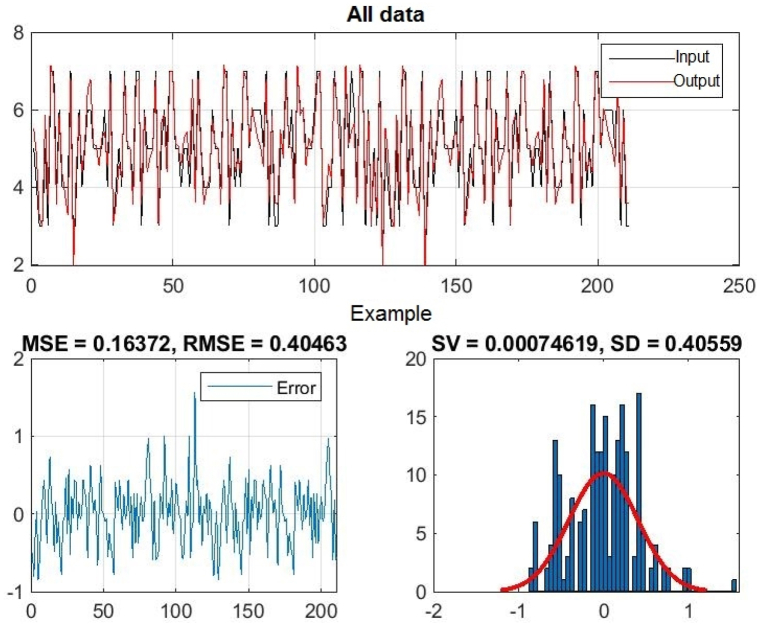
All data of the ANFIS network - two input.

**Fig. 11:**
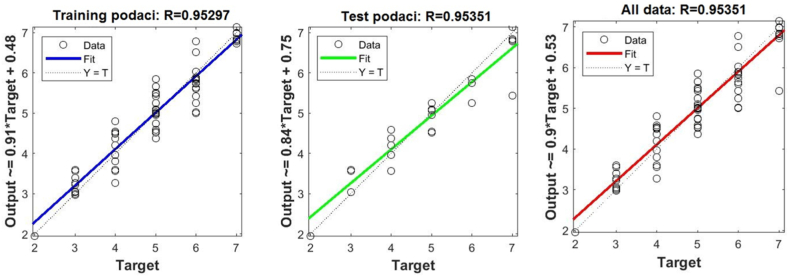
Regression of training, test, and all data - two inputs – achievements.

**Fig. 12:**
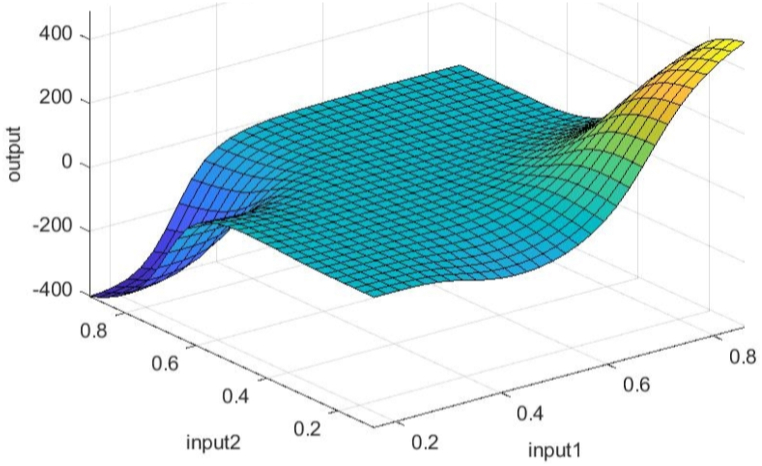
Graphical interpretation of training data - Matlab - two inputs - achievements.

Figs. 8, 9, and 10 represent training, test and all data obtained in the Matlab program and refer to errors during training and testing. Fig. 11 refers to the reliability of the model given through the regression analysis of training and test and all, and Fig. 12 to the approximation of the input data for training by the ANFIS output function.

In doing so, the effects of two combined inputs on the output were considered, and only the data for the combination of those inputs that have the greatest impact on the output variable, i.e., inputs 1 and 2, are shown.

In Fig. 8, in the upper part of the image, the training graph of Training data with the output of two achievement factors is shown, while in Fig. 9, in the upper part of the image, the graph of Test data is shown, as well as in Fig. 10, the graph of All data is shown. In the lower left corner, Fig. 8, Fig. 9, Fig. 10 show a graph with the obtained root mean square error MSE and root mean square error RMSE. While in the lower right corner, the graph of obtaining the results of the mean value of the error SV and the mean deviation SD is shown.

Fig. 11 shows the linear correlation coefficient - R for Training, Test and All data, the two most influential factors. And this coefficient is considered reliable because the value of the linear regression coefficient is greater than 0.8.

In the end, the influence of the three combined effects of the input variables on the output size is presented. Inputs 1, 2, and 4 have the smallest RMSE, so their joint influence on the output is the greatest, that is, the student's grade on the final exam is most influenced by these three factors together. Completing homework along with regular class attendance and independent engagement in the course can improve student achievement on the final exam in Introduction to Programming. The errors of training, testing and all data together when processing the three most influential input variables on the output, as well as the reliability coefficient of the obtained model, are presented in Table 4.

**Table 4:** The impact of three inputs on output - student achievement.

Input	The input with the least error
Three Input	Input no. 1–2 - 4
	TRAINING – ERROR
	SV = 0.000002 SD = 0.234181 MSE = 0.054534 **RMSE** = 0.233526
	TEST – ERROR
	SV = 0.095499 SD = 0.646684 MSE = 0.414251 **RMSE** = 0.643624
	ALL DATA
	SV = 0.014485 SD = 0.33075 MSE = 0.10909 **RMSE** = 0.33029
Relibility of the model	Training data: R = 0.98458
	Test data: R = 0.91624
	All data: R = 0.97029

**Fig. 13:**
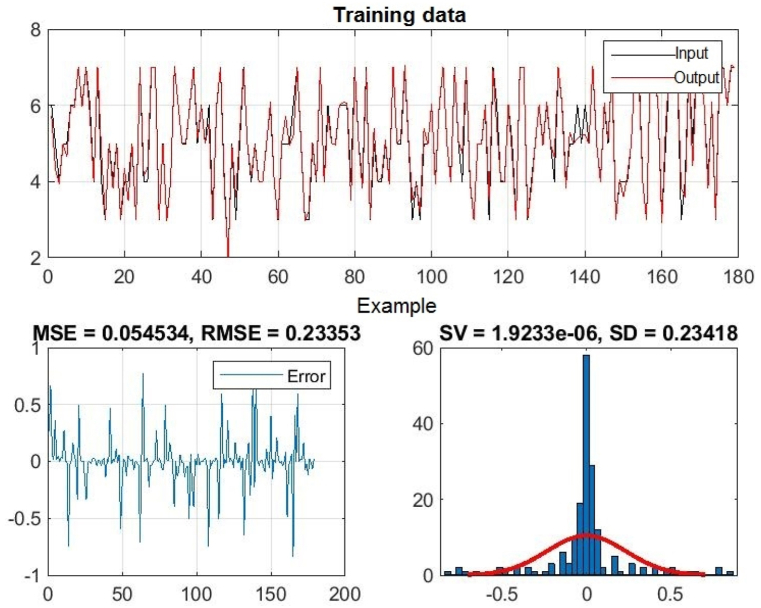
ANFIS network training - three inputs – achievements.

**Fig. 14:**
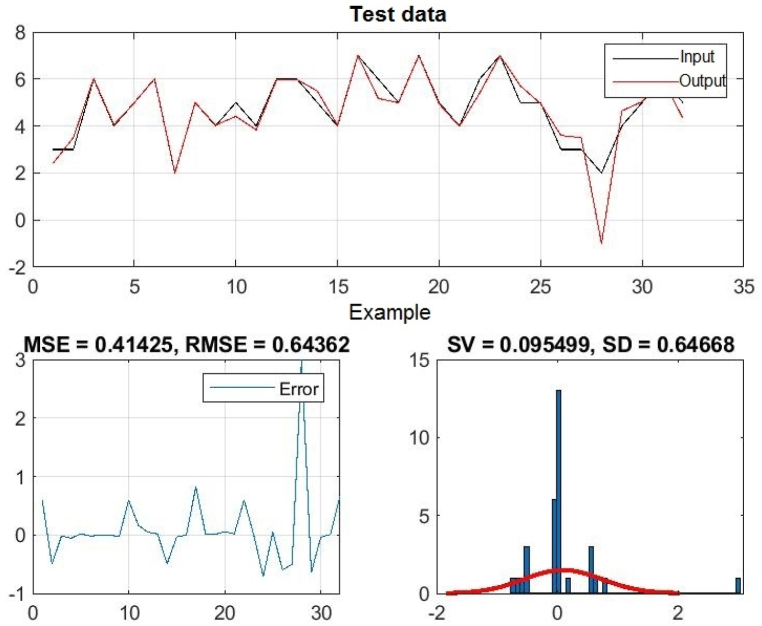
ANFIS network test - three inputs – achievements.

**Fig. 15:**
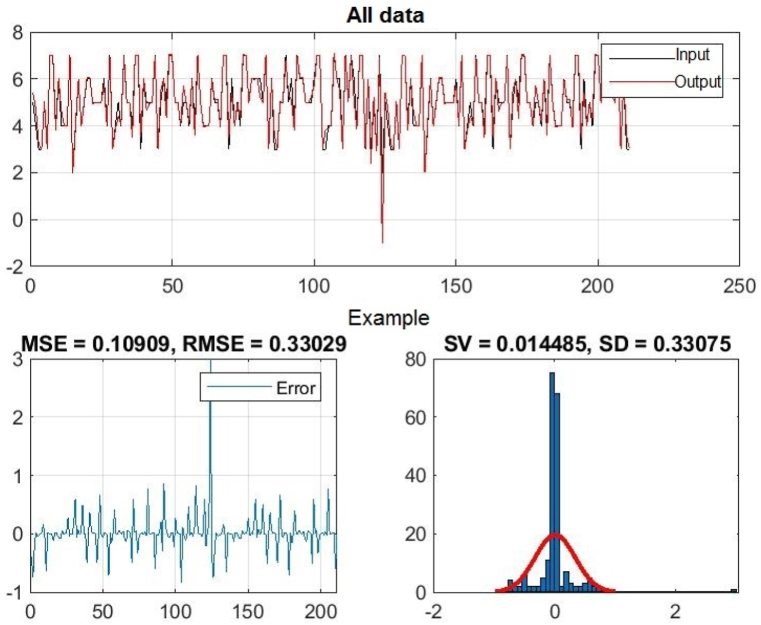
All data of the ANFIS network - three inputs – achievements.

**Fig. 16:**
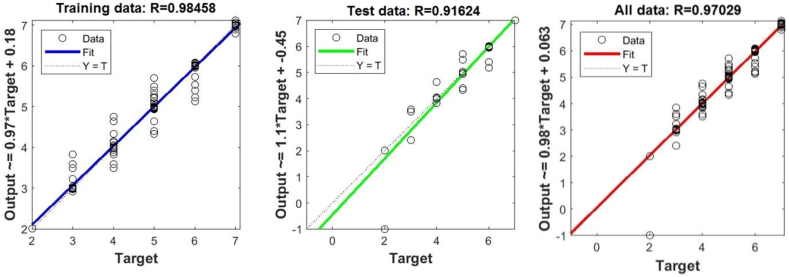
Regression of training, test and all data - three inputs – achievements.

**Fig. 17:**
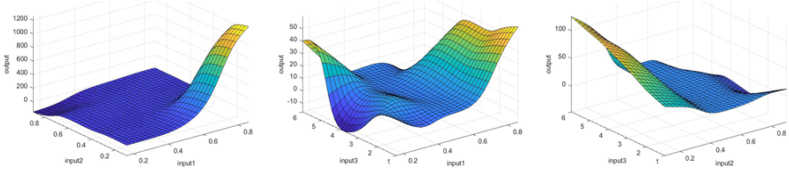
Graphical interpretation of training data - Matlab - three inputs – achievements.

Figs. 13, 14, and 15 show all the error data. Fig. 16, Fig. 17 show the graphical interpretation of the regression and approximation training and test of the three most influential inputs for the ANFIS output. In Fig. 13, in the upper part of the image, the training graph of Training data with the output of three achievement factors is shown, while in Fig. 14, in the upper part of the image, the graph of Test data is shown, as well as in Fig. 15, the representation of the graph of All data. In the lower left corner, Fig. 13, Fig. 14, Fig. 15 show a graph with the obtained mean square error MSE and root mean square error RMSE. While in the lower right corner, the graph of obtaining the results of the mean value of the error SV and the mean deviation SD is shown.

Fig. 16 shows the linear correlation coefficient - R for Training, Test and All data, the three most influential factors. Also, this coefficient is considered reliable because the value of the linear regression coefficient is greater than 0.8.

Fig. 17 shows approximations of the training, test and combined data of the three most influential inputs to the ANFIS output.

In this part of the research, using the Anfis method, it was examined in more detail which factors are part of the pre-examination requirements, which is the result of the previous study of the Random Forest algorithm, and which were divided into several input factors that have the greatest impact on the student's final grade.

The first research result obtained is that attendance at classes has the greatest influence on the final grade, which is logical, attendance at classes provides students with the opportunity to learn in an environment that is designed to adapt to their learning. Professors who teach the classes often use a variety of methods and techniques to help students understand programming concepts and techniques that are more difficult to learn outside of the classroom. Students who are present in class have the opportunity to interact directly with the professor, ask questions and discuss problems, which helps to better understand the material.

Two combined factors, class attendance and independent work, together have the greatest influence on the outcome. A combination of class attendance and independent work can bring the best results - regular class attendance and homework are key to understanding and mastering programming concepts. When these two factors are combined, students can achieve the best results in learning and the final grade.

Finally, the three combined factors that have the greatest impact are attendance at classes, independent work, and doing homework. Completing homework after students have actively participated in class and practiced at home can contribute to a better understanding and application of the material and thus contribute to achieving the best grade.

## 5 Conclusion

In this research, the influence of the first two input factors, prior knowledge and pre-examination obligations, on the achievement of students at vocational schools was examined, using the Random Forest algorithm, and then the obtained result was divided into several inputs to obtain a more detailed view of the influence of pre-examination obligations on student achievement. using the Anfis method.

The results of research using the Random Forest algorithm showed that pre-examination obligations are a key factor that affects student achievement, and the results of the Anfis method showed that attendance at classes, independent work and doing homework have the greatest influence on student success. The first result obtained by researching one factor that has the greatest impact on the achievement of students in the Introduction to Programming course is input 2, which is independent work. In addition to the obtained one, the biggest impact of input on output, an assessment of the impact of two factors that have the greatest impact on output was also carried out. It was found that input 1 and input 2 in combination have the smallest RMSE, i.e., the biggest impact on the output size, which means that attendance at classes and exercises and time spent in the independent study has the most influence on the final number of points, i.e. the grade at the end of the semester. In the end, the influence of the three combined effects of the input variables on the output size is shown. Inputs 1, 2, and 4 have the smallest RMSE, so their joint influence on the output is the greatest, that is, the student's grade on the final exam is most influenced by these three factors together. Completing homework along with regular class attendance and independent engagement in the course can improve student achievement on the final exam in Introduction to Programming. This practically means that the first hypothesis is confirmed, while the second one is not.

The results of this research confirm earlier research that also pointed to the importance of pre-exam duties in achieving good results on the exam. The limited set of input factors that focus on the analysis of input factors, which were selected by the author, many other factors affect the research and are not included in the paper.

The importance of this research is that it has been shown that the combination of various artificial intelligence methods can improve previous research in this area.

These findings can be useful for teachers to identify key factors that influence student achievement and thus adjust their teaching methods and approaches. Also, this research provides students with useful guidance on what are the key success factors in learning programming in vocational schools.

The key findings of this research, which deals with the creation of a statistical assessment of the achievements of professional students in the subject Introduction to Programming using a combination of the Random Forest algorithm and the ANFIS method, include the use of the Random Forest algorithm in the analysis, which proved to be a powerful tool for identifying factors that affect student achievement. Also, the application of the ANFIS method contributed to a better understanding of the complex interactions between input factors and output variables.

Importance of Optimization Algorithms show deviations in the results that are a consequence of the random distribution of data. However, it is emphasized that algorithm optimization can help reduce these deviations and improve model accuracy.

These results have practical implications for the educational system and the vocational education system. For example, schools could focus on providing additional support to students in terms of exam preparation and pre-examination tasks to improve their exam performance.

Further improvement of this research would be the use of other methods of artificial intelligence and the comparison of the obtained results to obtain the most optimal solution to achieve better student success, both in this and in other teaching subjects.

## Data availability

Data will be made available on request.

## CRediT authorship contribution statement

**Marija Mojsilović:** Conceptualization, Methodology, Writing – original draft, Writing – review & editing. **Radoje Cvejić:** Conceptualization, Methodology, Writing – original draft, Writing – review & editing. **Selver Pepić:** Conceptualization, Methodology, Writing – original draft, Writing – review & editing. **Darjan Karabašević:** Methodology, Supervision, Writing – original draft, Writing – review & editing. **Muzafer Saračević:** Conceptualization, Methodology, Writing – original draft, Writing – review & editing. **Dragiša Stanujkić:** Conceptualization, Methodology, Writing – original draft, Writing – review & editing.

## Declaration of competing interest

The authors declare that they have no known competing financial interests or personal relationships that could have appeared to influence the work reported in this paper.
